# Evaluation of the normal-to-diseased apparent diffusion coefficient ratio as an indicator of prostate cancer aggressiveness

**DOI:** 10.1186/1471-2342-14-15

**Published:** 2014-05-10

**Authors:** Andrei Lebovici, Silviu A Sfrangeu, Diana Feier, Cosmin Caraiani, Ciprian Lucan, Mihai Suciu, Florin Elec, Gheorghita Iacob, Mircea Buruian

**Affiliations:** 1 Radiology Department, Emergency County Hospital, “Iuliu Hatieganu” University of Medicine and Pharmacy, Cluj-Napoca, Romania; 2 Department of Imaging, Regional Institute of Gastroenterology and Hepatology, “Iuliu Hatieganu” University of Medicine and Pharmacy, Cluj-Napoca, Romania; 3 Department of Urology, Clinical Institute of Urology and Kidney Transplant, “Iuliu Hatieganu” University of Medicine and Pharmacy, Cluj-Napoca, Romania; 4 Department of Pathology, Clinical Institute of Urology and Kidney Transplant, “Iuliu Hatieganu” University of Medicine and Pharmacy, Cluj-Napoca, Romania; 5 Department of Radiology, County Emergency Hospital Mures, University of Medicine and Pharmacy, Targu-Mures, Romania

**Keywords:** Prostate cancer, Diffusion-weighted magnetic resonance imaging, Neoplasm grading

## Abstract

**Background:**

We tested the feasibility of a simple method for assessment of prostate cancer (PCa) aggressiveness using diffusion-weighted magnetic resonance imaging (MRI) to calculate apparent diffusion coefficient (ADC) ratios between prostate cancer and healthy prostatic tissue.

**Methods:**

The requirement for institutional review board approval was waived. A set of 20 standardized core transperineal saturation biopsy specimens served as the reference standard for placement of regions of interest on ADC maps in tumorous and normal prostatic tissue of 22 men with PCa (median Gleason score: 7; range, 6–9). A total of 128 positive sectors were included for evaluation. Two diagnostic ratios were computed between tumor ADCs and normal sector ADCs: the ADC peripheral ratio (the ratio between tumor ADC and normal peripheral zone tissue, ADC-PR), and the ADC central ratio (the ratio between tumor ADC and normal central zone tissue, ADC-CR). The performance of the two ratios in detecting high-risk tumor foci (Gleason 8 and 9) was assessed using the area under the receiver operating characteristic curve (AUC).

**Results:**

Both ADC ratios presented significantly lower values in high-risk tumors (0.48 ± 0.13 for ADC-CR and 0.40 ± 0.09 for ADC-PR) compared with low-risk tumors (0.66 ± 0.17 for ADC-CR and 0.54 ± 0.09 for ADC-PR) (p < 0.001) and had better diagnostic performance (ADC-CR AUC = 0.77, sensitivity = 82.2%, specificity = 66.7% and ADC-PR AUC = 0.90, sensitivity = 93.7%, specificity = 80%) than stand-alone tumor ADCs (AUC of 0.75, sensitivity = 72.7%, specificity = 70.6%) for identifying high-risk lesions.

**Conclusions:**

The ADC ratio as an intrapatient-normalized diagnostic tool may be better in detecting high-grade lesions compared with analysis based on tumor ADCs alone, and may reduce the rate of biopsies.

## Background

The assessment of local aggressiveness of prostate cancer (PCa) is of key importance for appropriate management of this disease. The increase in life expectancy of the general population combined with efficient screening methods will lead to an increase in the number of new PCa cases [1]. These cases will tend to be more localized and at an earlier stage. Over the last few years, new focal methodologies have emerged for PCa treatment, such as high-intensity focused ultrasound, cryotherapy, focal laser ablation, intensity-modulated radiation therapy, radiofrequency ablation, and others. These relatively new therapeutic methods come as alternatives to existing treatment modalities such as radical prostatectomy or radiotherapy [2]. The diagnosis of PCa is based on physical (i.e., digital rectal) examination, prostate specific antigen (PSA) levels, and, when PSA levels are abnormal, blind biopsy. All of these assessments are of low diagnostic accuracy [3-5]. Multiparametric magnetic resonance imaging (MRI) has shown great potential, with significant improvement in detection, localization, and characterization of PCa [6,7]. This is of great importance for the selection of precise treatment options, and for minimizing treatment-related morbidity. Patients with tumors rated with a Gleason score of 6 or greater represent an important target population for PCa detection and effective therapy [8,9].

According to recent guidelines [10], multiparametric MRI of the prostate includes three functional MR sequences: diffusion weighted imaging (DWI) [11], dynamic contrast enhancement-MR [12], and MR spectroscopy [13]. These sequences contribute to the PI-RADS scoring system of prostate lesions [10], with DWI as the main contributor. With a short acquisition time and high contrast resolution between tumor and normal prostatic tissue [14], DWI and apparent diffusion coefficient (ADC) mapping demonstrate an inverse and significant correlation with the Gleason score [7,14]. Although ADC can estimate tumor aggressiveness, there is still a large overlap between ADCs of different tumors with the same Gleason score [7]. The purpose of this study was to establish a simple method for PCa assessment of tumor aggressiveness using ADC ratios between prostate cancer and healthy prostatic tissue.

## Methods

We analyzed the images of 43 men who underwent prostate MRI examinations with an endorectal coil in our institution between February 2011 and August 2012. Written informed consent had been obtained from patients prior to the MR examinations, as well as before all interventions, such as prostatic biopsy. The requirement to obtain written, informed consent for the retrospective analysis of the data was waived. To be included in our study, subjects needed to have biopsy-proven PCa from a standard 20-core transperineal saturation biopsy. We excluded five cases because of post-biopsy hemorrhage, five cases because of motion artifacts, and 11 cases because of unavailable or negative biopsy specimens. Thus, 22 cases (median age: 64.5 years; range: 52–75 years) were further selected in this retrospective single-center study. All subjects had undergone MRI with an endorectal coil including T2-weighted images (T2WI) and DWI 4–6 weeks after PCa confirmation by biopsy.

### Saturation biopsy

**Figure 1:**
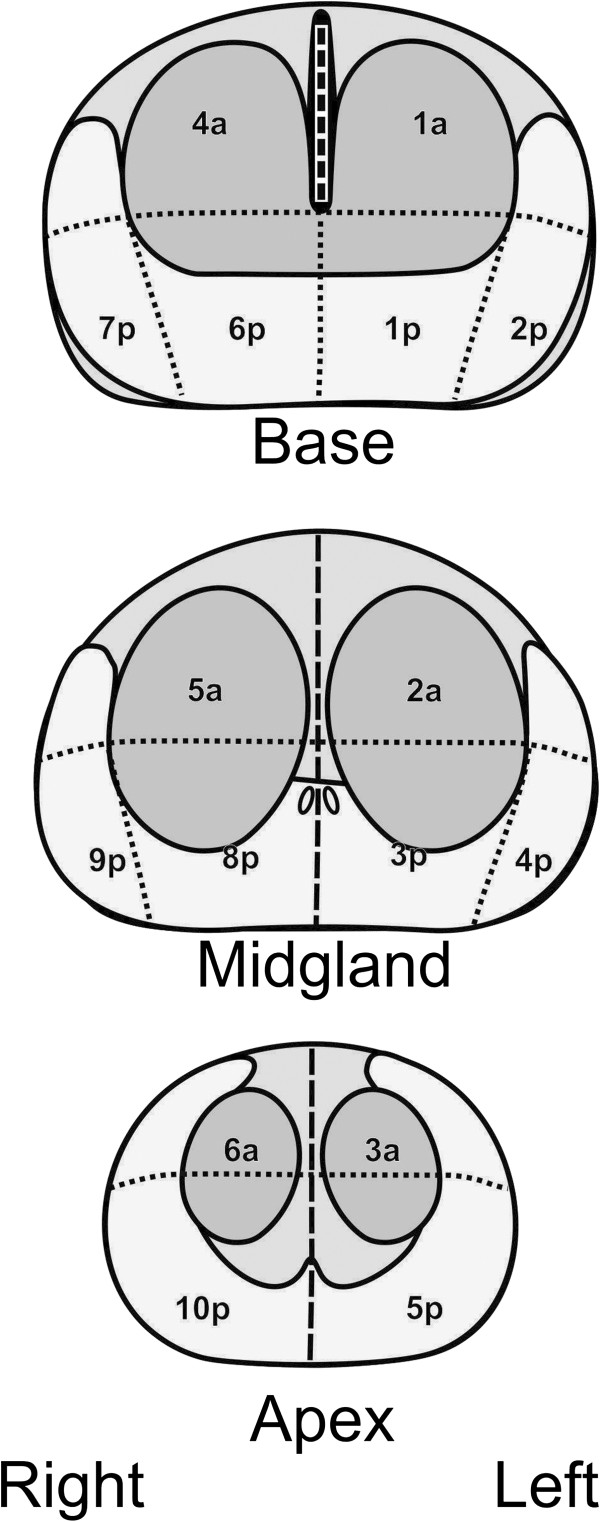
Prostate segmentation showing ten posterior and six anterior segments.

Saturation biopsy was conducted under general anesthesia in the supine position. The biopsy needle was guided with transrectal ultrasound into the sectors (Figure 1) and 20 transperineal cores were obtained. Each biopsy core was numbered, assigned to a sector, and sent for histopathological analysis.

### Histopathological analysis

Biopsy specimens were fixed in formaldehyde, embedded in paraffin, and stained with hematoxylin-eosin. The diagnosis was confirmed with immunohistochemistry for basal cells (p63) and AMCAR expression (p504s). Results were reported as cancer with an assigned Gleason score or as benign tissue.

### MRI technique and image interpretation

MRI was performed with a 1.5 T scanner (Symphony; Siemens AG, Erlangen, Germany) using an eight-channel phased array body coil combined with an endorectal coil (MEDRAD, Inc., Warrendale PA, USA). After digital rectal examination, the balloon of the endorectal coil was inflated with 60 mL of air. T2WI were obtained in axial, coronal, and sagittal planes using turbo spin echo sequences and the entire prostate was investigated. DWI was performed in the axial plane using echo-planar imaging (EPI) sequences at three b-values (0, 400, and 800 mm^2^/s) and restriction of diffusion was quantified by ADC values. T2WI parameters and DWI parameters are shown in Table 1.

**Table 1:** MRI parameters’ description

	**T2W-MRI**	**DW-MRI**
Sequence	Fast spin echo	Spin echo EPI
TR (ms)	5500	3200
TE (ms)	104	90
Flip angle	150°	
FOV (mm^2^)	180 × 180	300 × 300
Matrix	256 × 256	128 × 128
Voxel size (mm^3^)	0.8 × 0.8 × 3	2.3 × 2.3 × 4
Slice thickness (mm)	3	4
Gap (mm)	0.3	0.3
Spectral suppression	No	Yes
b-values	-	0/400/800
NEX	1	2
TA (min:sec)	4:09	2:40

### Image interpretation

**Figure 2:**
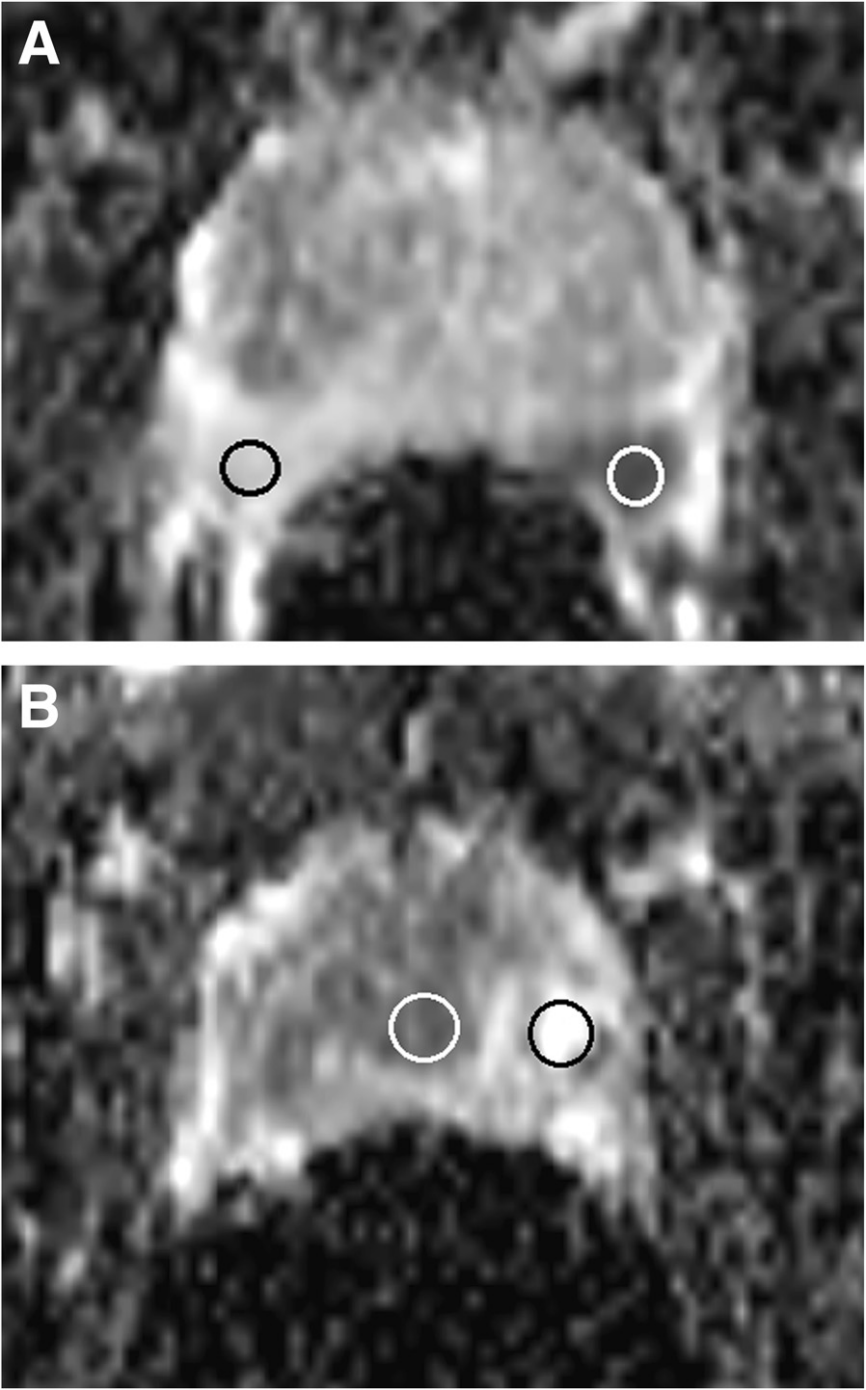
**Placement of regions of interest (ROI) for (A) peripheral gland tumor (B) central gland tumor. A**. Placement of regions of interest (ROI) for signal intensity measurements of Gleason 8 (4 + 4) peripheral tumor (white ROI = 0.78 × 10^-3^ mm^2^/s) and normal peripheral zone (black ROI = 1.9 × 10^-3^ mm^2^/s). The ADC-PR (white ROI/black ROI) is 0.41, which indicates a high-grade tumor. **B**. The white ROI is placed in the central gland (CG) portion of a Gleason 9 (4 + 5) lesion (0.82 × 10^-3^ mm^2^/s) while the black ROI is located in tumor-free CG tissue (1.87 × 10^-3^ mm^2^/s) The ADC-CR (white ROI/black ROI) is 0.43, which indicates a high-grade prostate tumor.

Image interpretation was done on a PACS station (KODAK/Carestream Version 10.2; Carestream Health, Rochester, NY, USA) by two radiologists with 5 years’ combined experience in uroradiology and prostate imaging and 3 years’ combined experience interpreting DWI (L.A., C.C.). The readers were aware of the results of biopsy and clinical data. PCa-positive biopsy cores were assigned to a 16-region standardized prostate reporting scheme including ten posterior and six anterior glandular sectors (Figure 1) as recommended by Dickinson et al. [15]. ADC maps were generated from the DWI sequences, and regions of interest (ROIs) were placed according to biopsy results and the prostate reporting scheme. Lesions with low signal intensity compared with surrounding tissue on ADC were considered malignant. ROIs were drawn to occupy approximately 75% of the lesion in a given segment to be sure that normal tissue outside lesion margins would not be included. ROIs were drawn also in the tumor-free sectors, with a standard ROI size of 0.8 cm^2^. The mean signal intensity was measured automatically by the PACS system. ADC ratios were calculated by dividing tumoral ADCs by tumor-free ADCs from the peripheral and the central zones of the gland (Figure 2A and B). The highest ADCs of normal sectors were taken as reference values. T2WI and T1-weighted images (T1WI) were assessed for post-biopsy hemorrhage.

### Statistical analysis

The analysis was performed per patient and per tumor unit accordingly. Categorical variables were presented as numbers and percentages. Continuous variables were expressed as mean values and standard deviation or median and range, as appropriate. We used a linear multilevel model, so no linearity or normality assumptions were made. The discriminative capability of each ADC ratio was determined by calculating the area under the receiver operating characteristic curve (AUC). Optimal cut-off values were chosen to maximize the Youden index, and sensitivity (%) and specificity (%) were computed from the same data, without further adjustments. Discriminative capability was examined per tumor unit and not per patient, because of the low number of available patients. Comparisons were performed among different ADCs using the Student’s *t*-test for continuous variables and p < 0.05 was considered statistically significant. The statistical analysis was performed using commercially available software (MedCalc version 12.4.0; MedCalc Software, Mariakerke, Belgium).

## Results

Median PSA serum levels were 15.5 ng/mL (range, 6.8–100 ng/mL) and median prostate volume was 44 mL (range, 31–67 mL). On saturation biopsy, cancer was detected in 157 of the 440 cores (36%). After assigning the cancer-positive cores to the 16-region standardized prostate reporting scheme, 128 out of 352 regions were cancer positive (36%). The median Gleason score was 7 (range: 6–9). Gleason score 6 tumors were detected in 12 regions, Gleason score 7 tumors were detected in 38 regions, Gleason score 8 tumors were detected in 31 regions, and Gleason score 9 tumors were detected in 46 regions. From the positive tumor sectors, 86 (67%) were peripherally located, while 42 (33%) were centrally located.

### ADC findings

Results of mean prostatic tumor ADC for lower-grade cores (Gleason 6 and 7) and higher-grade cores (Gleason 8 and 9) were significantly different (p < 0.001), as were the ADC-PR and ADC-CR (p < 0.0001), as presented in Table 2.

**Table 2:** ADC and ADC-R characteristics of 128 prostatic sectors for low- and intermediate-risk group (Gleason 6 and 7) and high-risk group (Gleason 8 and 9)

**Biopsy specimen characteristics**	**Gleason 6 and 7 (n = 51)**	**Gleason 8 and 9 (n = 77)**	**p**
*Number of patients	14 (64%)	8 (36%)	
Age (years)	66 (56–76)	59 (46–76)	<0.001
PSA (ng/mL)	10.74 (3.5-25)	26 (14–100)	<0.001
ADC (mm^2^/sec)	0.88 × 10^-3^ ± 0.13	0.73 × 10^-3^ ± 0.17	<0.001
Normal central zone ADC (mm^2^/sec)	1.38 × 10^-3^ ± 0.24	1.59 × 10^-3^ ± 0.22	<0.001
Normal peripheral zone ADC (mm^2^/sec)	1.64 × 10^-3^ ± 0.19	1.90 × 10^-3^ ± 0.35	<0.001
ADC-CR	0.66 ± 0.17	0.48 ± 0.13	<0.001
ADC-PR	0.54 ± 0.09	0.41 ± 0.09	<0.001

Note: Data are presented as mean and standard deviation or median and range, except where indicated. *: Data are presented as number and percentages. ADC-CR: ratio between tumor ADCs and healthy central gland tissue ADCs. ADC-PR: ratio between tumor ADCs and healthy peripheral zone tissue ADCs.

For a cut-off level ≤ 0.82 ± 0.32 × 10^-3^ mm^2^/s, the tumor ADCs for Gleason 8 and 9 positive sectors showed a sensitivity and specificity of 72.7 and 70.6%, while the AUC was 0.75. Using a cut-off value ≤ 0.57, the ADC-CR showed more promising results with sensitivity = 82.2%, specificity = 66.7%, and AUC of 0.77 (Table 3). The best diagnostic performance was obtained for ADC-PR, with an AUC of 0.84, when applying a cut-off value ≤ 0.5.

**Table 3:** DWI cut-off values and performance values of tumor ADC, ADC-CR, and ADC-PR in predicting Gleason 8 and 9

	**Tumor ADC**	**ADC-CR**	**ADC-PR**
Cut-off	≤ 0.82	≤ 0.57	≤ 0.5
Sensitivity	72.7	82.2	88.9
Specificity	70.6	66.7	64.7
**AUROC**	**0.75**	**0.77**	**0.84**
**p value**	**< 0.001**	**< 0.001**	**< 0.001**

Note: AUC: area under the receiver operating characteristic curve. ADC-CR: ratio between tumor ADCs and healthy central gland tissue ADCs. ADC-PR: ratio between tumor ADCs and healthy peripheral zone tissue ADCs.

**Figure 3:**
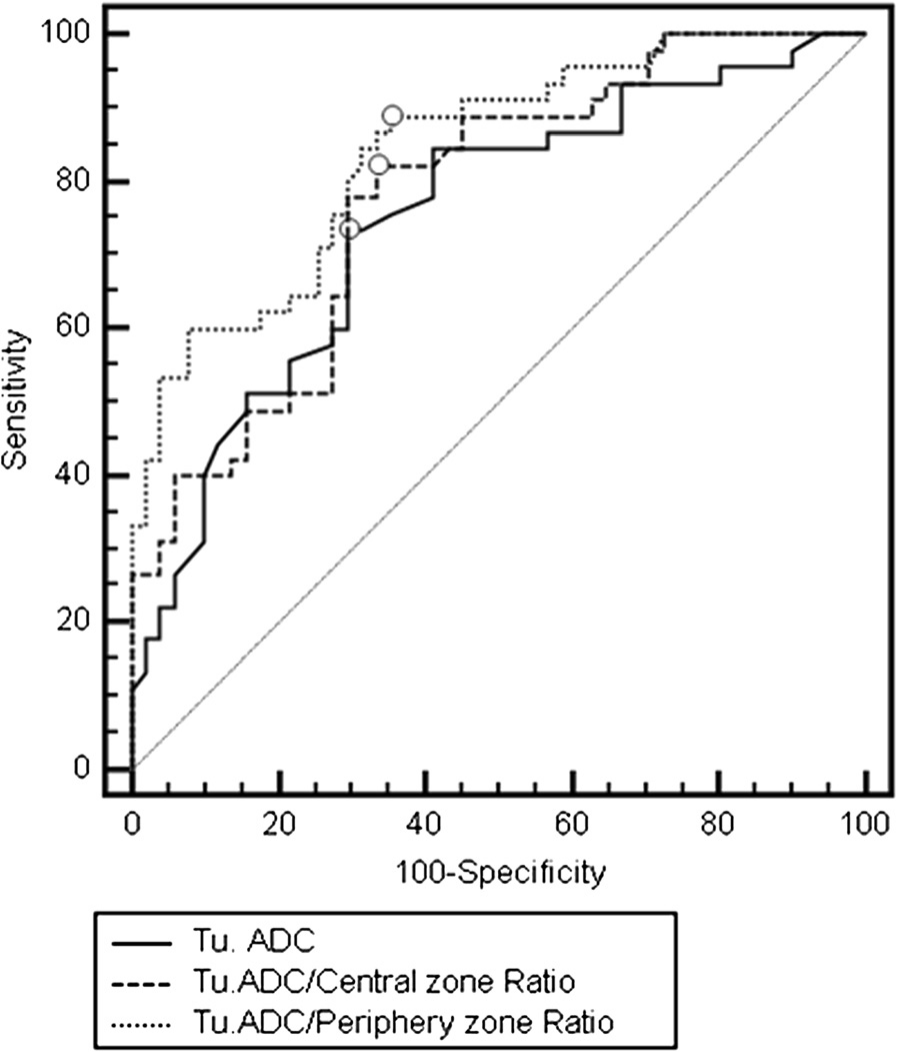
Comparison of receiver operating characteristic curves for tumor ADC, ADC-CR, and ADC-PR for cases with Gleason scores 8 and 9.

When analyzing the performance for ADC-PR in detecting tumors with a high Gleason score (8 or 9) in relation to the lesion location, the ratio had robust accuracy in detecting central zone tumors (AUC = 0.81) and an excellent discriminatory ability to detect tumors located in the peripheral zone (AUC = 0.90) (Figure 3). The results are summarized in Table 4.

**Table 4:** The cut-off values and diagnostic performance of ADC-Peripheral Ratio in predicting Gleason 8 and 9 stages, according to tumor location

	**Central zone tumors**	**Peripheral zone tumors**
Cut-off	≤ 0.42	≤ 0.50
Sensitivity	62.1	93.7
Specificity	92.7	80
**AUC**	**0.81**	**0.90**
**p value**	**< 0.001**	**< 0.001**

Note: AUC: area under the receiver operating characteristic curve.

## Discussion

Our study shows promising results in the assessment of PCa aggressiveness using tumor-to-normal ADC ratios. In this study, both central and peripheral ADC ratios demonstrated a better sensitivity, specificity, and AUC than the interpretation of ADCs alone, with the most promising results obtained for the ratios computed using normal ADCs measured in the periphery of the gland. It is not yet possible to find a cut-off value to delineate malignant tissue from benign because of the overlap of ADCs of PCa and those of normal prostatic tissue; even Gleason 9 tumors showed ADCs up to 1.27 × 10^-3^ mm^2^/s. This finding is well known; Litjens et al. concluded in their study that tumor ADCs should not be considered absolute because of the influence of “background” variation of normal peripheral zone (PZ) tissue composition [16]. This statement is supported by the results of our study, in which we were able to demonstrate better AUC for ADC ratios in comparison with tumor ADCs alone. In our study, the ADCs of the PCa-positive biopsy cores showed statistically significant differences from the biopsy cores of normal PZ and central gland (CG). These results are consistent with results previously published [17-19]. The mean ADC for positive PCa cores in our group (0.79 ± 0.18 × 10^-3^ mm^2^/s) was slightly higher than the one reported by Woodfield et al. [14] (0.737 ± 0.154 × 10^-3^ mm^2^/s, range, 0.108–1.263 × 10^-3^ ) but lower than results published recently by Yamamura et al. [18] (0.96 ± 0.24 × 10^-3^ mm^2^/s), Rinaldi et al. [19] (0.99 ± 0.15 × 10^-3^ mm^2^/s) and Nagayama et al. [20] (1.07 ± 0.35 × 10^-3^ mm^2^/s) (p > 0.5). The lower ADCs for positive cores in our study group were probably because of the high number of Gleason score 8 and 9 biopsy-proven sectors, which exceeded 60% of all PCa positive biopsies. This large number of biopsy-proven, high-grade PCa is likely the consequence of larger tumors in this group of patients and a higher percentage of positive biopsy cores (for example, all 20 biopsy cores were tumor-positive in two patients).

Healthy PZ prostatic tissue is rich in tubular structures, allowing water molecules to move easily. As a consequence, the ADCs of healthy PZ are high. Published ADC mean values for PZ have been 1.65 ± 0.21 × 10^-3^ mm^2^/s and 1.73 ± 0.27 × 10^-3^ mm^2^/s [15,18]. Our study of the normal PZ shows similar results, with a mean ADC of 1.71 ± 0.32 × 10^-3^ mm^2^/s. Normal CG structure is composed of two different tissues, a glandular component with high signal intensity on T2WI and a stromal component with low signal intensity on T2WI, which can mimic PCa. The ADCs of normal CG should be lower than normal values from the PZ because of benign stromal hyperplasia changes taking place in the central gland. Oto et al. showed that there is a significant difference between ADCs of hyperplasic stromal nodules (1.27 ± 0.21 × 10^-3^ mm^2^/s) and hyperplastic glandular nodules (1.73 ± 0.28 × 10^-3^ mm^2^/s) [21]. Our measurements were done on the entire CG including both macroscopically stromal and glandular areas and the mean ADC was 1.44 ± 0.22 × 10^-3^ mm^2^/s, which is lower than the PZ ADC and close to the results published by Oto et al. [22].

When comparing the Gleason scores, the ADC values in subjects with Gleason 6 and 7 were significantly lower than those with Gleason 8 and 9. As an additional observation, patients with Gleason 8 and 9 were significantly younger (p < 0.0001). We hypothesize that the ADC differences arise from natural variations in prostate physiology in younger patients, owing to less significant fibrotic and atrophic changes of prostate glandular structure that would tend to decrease the ADC measurements [17].

In the last several years, many authors have investigated the correlation between ADC and Gleason score. Woodfield et al. [14] demonstrated a statistically significant difference between ADCs of low-grade tumors (Gleason 6) and intermediate-grade tumors (Gleason 7) and between low-grade tumors and high-grade tumors (Gleason 8 and 9). Verma et al. [7], in a large study of 197 prostate tumors, concluded that ADCs negatively correlated with the Gleason score. Somford et al. [22] showed the capacity of ADC mapping to predict the presence of high-grade tumors in patients with Gleason 6 (3 + 3) findings on biopsy. Although there are promising results with DWI, there are still discrepancies between ADCs of PCa and the final Gleason score in the many papers published. The differences among ADCs can be explained by physiologic factors such as age and tumor size and by technical factors (e.g., acquisition parameters, software, and the use of endorectal coils). The results of our study are very encouraging; we demonstrated that there is a statistically significant difference between ADCs of low- and intermediate-grade tumors compared with high-grade tumors. Our results are consistent with those reported by Woodfield et al. [14].

PCa is a major health problem. The diagnostic methodology remains practically the same, with ultrasound-guided systematic biopsy as the main diagnostic modality. Systematic biopsy has important limitations; it can miss up to 35% of cancers [23], and the Gleason score resulting from systematic biopsy has to be upstaged in up to 50% of the patients after radical prostatectomy [24]. In addition, Nam et al. concluded in their extensive study that complications after TRUS-guided biopsy have increased dramatically in the last 10 years [25]. With, new less-invasive focal therapy techniques and the active surveillance approach (which is based only on biopsy results), it is of great importance to improve PCa detection, characterization, and staging while reducing biopsy-related morbidity as much as possible.

The results of this study show the potential of ADC mapping in estimating the aggressiveness of PCa. By analyzing the ADC ratios when applying DWI sequences in PCa imaging, we can detect high-grade tumors more accurately than when carrying out analysis based on ADC alone. From our point of view, ADC mapping has the potential to guide the biopsy needle into the most aggressive region of a malignant lesion, resulting in fewer and more accurate biopsies, better staging, and more information to select appropriate treatment options.

We have to state several limitations: (1) We used a systematic biopsy as the reference standard, even though it may miss a substantial percentage of prostate cancers. However, it is the method of choice for cancer detection. (2) Our study population was relatively small. Patients with Gleason 8 and 9 scores tend to be younger than patients with Gleason 6 and 7 scores, so the cases could not be age-matched. (3) We do not have data on intraobserver and interobserver variability. (4) Prostate biopsy was performed prior to MRI examination, which might have influenced the ADC measurements. The presence of microscopic hemorrhage undetectable on standard MRI sequences might have altered the ADC values and underestimated the diagnostic value of ADC ratios.

## Conclusions

The ADC tumor-to-normal ratio may be more predictive of tumor grade than analysis based on ADCs alone.

## Abbreviations

PCa: Prostate cancer; ADC: Apparent diffusion coefficient; ADC-PR: Ratio of tumor ADC to normal PZ ADC; PZ: Peripheral zone; CG: Central gland; ADC-CR: Ratio of tumor ADC to normal CG ADC; PSA: Prostate specific antigen; MRI: Magnetic resonance imaging; T2WI: T2-weighted images; T1WI: T1 weighted images; DWI: Diffusion weighted imaging; AUC: Area under receiver operating characteristic curve.

## Competing interests

The authors declare that they have no financial or non-financial competing interests.

## Authors’ contributions

LA performed the study concept and design, acquisition of data, analysis and interpretation of data, and drafted the manuscript. SS supervised the study concept and design and helped draft the manuscript. FD formulated the study concept and design, analysis, and interpretation of data, and drafting of the manuscript. CC performed the imaging acquisition, interpretation of data, and helped draft the manuscript. LC performed the acquisition of data and drafting of the manuscript. SM performed analysis and interpretation of data and drafting of the manuscript. EF performed the acquisition of data and helped draft the manuscript. IG performed the histological studies and analysis of data. BM performed critical revision of the manuscript for important intellectual content. All authors read and approved the final manuscript.

## Pre-publication history

The pre-publication history for this paper can be accessed here:

http://www.biomedcentral.com/1471-2342/14/15/prepub
